# Medical History from the Earliest Times

**Published:** 1892-05-14

**Authors:** E. T. Withington


### Medical History from the Earliest Times.

By E. T. Withington, M.A., M.B. Oxon.

### VIII.—THE EARLIEST GREEK MEDICINE—HOMER—ÆSCULAPIUS.

Among the lost treatises of Galen is one "On the Practice of Medicine in Homer," a subject which has since been very frequently discussed in works ranging from Daremberg's "La Médecine dans Homère," where every word in the poems of possible medical significance is considered, down to monographs, such as Fröhlich's "Hygienic Thoughts on the Chiton of the Homeric Heroes," which appeared in that serious work, Virchow's "Archiv," and was preceded by papers on the medical aspects of "Barracks During the Trojan War," and "The Head-dress of the Homeric Heroes," titles reminding us of the famous German feat of evolving a camel from the inner consciousness. For Homer, though he does not, like a great mediæval poet, professedly omit all medical details as unsuited to polite ears, treats the subject to some extent from a poetic standpoint. His wounded heroes either die at once, or, after very simple treatment, return to the fight as vigorous as before. There are no cases of traumatic or inflammatory fever, and no one dies from secondary hæmorrhage; while, except the Plague, which forms an essential part of the story, there is no mention of those epidemics which have always formed the scourge of camps. But all this is surely to be attributed, not to the hygienic advantages of scanty dress and well-ventilated huts, but to the fact that descriptions of disease are not suited to epic poetry. Ordinary disease is seldom noticed, the chief passages being Od. v., 395, where the joy of Ulysses at the sight of land is compared to that of sons who see their father recovering from a long illness, in which an angry god assailed him; and ix. 411, where the blinded and howling Cyclops is told by his friends that, if he is ill, he should remember that sickness comes from Zeus, and is unavoidable. It is interesting to note that in both cases we find a supernatural theory of disease. Though a knowledge of drugs is the chief mark of a physician internal medicines are rarely mentioned, the word "pharmakon" denoting in the Iliad outward applications only, while in the Odyssey it means either a poison or a charm, such as those of Circe. Indeed, Helen's nepenthe seems to be the nearest approach to what we should call a medicine, for the mixture of Pramnian wine with cheese and barley-meal, which Nestor gave the wounded Machaon, appears to have been an ordinary form of food, since Nestor takes it himself, and, with the addition of honey, it forms the "mess" which Circe set before the sailors of Ulysses, and in which she placed her baleful pharmaka.

The most prominent part of Homeric medicine is naturally the treatment of wounds. Everyone seems able to render aid in emergencies; thus, when Ulysses is wounded by a boar, his comrades skilfully bind the wound and recite the appropriate incantations; but there is already a special class of men, skilled in removing embedded weapons, who know the drugs which stop bleeding and lessen pain. There are "the physicians skilled in medicines" who look after the wounded, and an equal or still higher knowledge is possessed by certain chiefs, Achilles, Patroclus, and, above all, the two sons of Æsculapius, "the cunning leeches" Podalirius and Machaon. The social position of these "Iatroi" is a very respectable one; in war they are "worth many other men," in peace they are, like the seer and the minstrel, welcome guests in the halls of the heroes. It is important to notice that there is no trace of any connection between this medical class and priests: if Calchas is called upon in the plague, it is purely in his capacity of seer; some god is angry; who he is, and how to appease him, are clearly theological not medical questions. Besides physicians, women have a knowledge of drugs: thus Nestor distinguishes a hero whom he slew in his youth, by saying "he had to wife the fair-haired Agamedê (the very wise) who knew all drugs, so many as the wide earth nourisheth." Circe and Helen have already been mentioned. As final examples of Homeric medicine we may mention the revival of Hector when struck down by Ajax, by means of copious douching with cold water; a truly heroic method of treating collapse: while Ulysses after the slaughter of the Suitors, purifies his house by burning sulphur in the most approved modern fashion.

Æsculapius (Asklepios), as everyone knows, is the god of medicine, but in Homer he is merely a Thessalian chieftain, who, like Achilles, has learnt from Chiron the knowledge of drugs, and has taught it to his sons, just as Achilles taught Patroclus. But Arctinus of Lesbos (B.C. 770), the first Greek poet, of whose personality we are certain, already invests him with supernatural attributes, and tells us that "he endowed one of his sons with nobler gifts than the other; for while to the one (Machaon) he gave skilful hands to draw out darts, make incisions, and heal sores and wounds, he placed in the heart of the other (Podalirius) all cunning to find out things invisible, and cure that which healed not," a passage in which medicine and surgery are clearly distinguished, and precedence given to the former.

It was probably about this time that temples began to be erected to the god of medicine; and in these "Asclepieia," which became very numerous (over 300 being mentioned by classic writers), there developed a peculiar form of medical treatment known as "incubation." Traces of this practice are to be found in ancient Egypt, but our earliest and most important notice of it in Greece is in the Plutus of Aristophanes (B.C. 388). The sick person after sacrifice and purification lay down to sleep near the altar of the god, and the mode of treatment was revealed to him either in a dream, or more directly by the priest himself, dressed so as to represent the deity. On recovery, he presented thank-offerings, sometimes including models of the affected part in wax, silver, or even gold (thus the temple at Athens possessed, among other treasures, a silver heart, gilded legs, gold and gilded eyes, &c.), and a tablet was put up describing his illness and its treatment. According to Pliny and Strabo, Hippocrates was much indebted to the tablets thus accumulated in the Asclepieion at Cos, and modern writers have even made the "Father of Medicine" himself a priest of Æsculapius. This latter point will be discussed when we come to speak of the Asclepiadæ; but the medical importance of the temple records has probably been much exaggerated. Unfortunately no ancient writer has thought it worth while to copy any of them, and—excepting the curious inscriptions recently discovered at Epidaurus—we are limited to three or four examples, of late date and doubtful authenticity, found at Rome. The cures there described are of a semi-miraculous nature, and the treatment, naturally, such as would connect them with the divine influence of the god. They differ from the histories of cases given by Hippocrates in every possible way, and if the inscriptions at Cos at all resembled them, they can only have been useful to him as warning examples of what a clinical history should not be. The following may serve as a specimen: "Julian being in a hopeless state on account of a spitting of blood, was directed by the god to take pine-seeds from the altar, mix them with honey, and eat them during three days. He recovered, and returned thanks openly before the people."

But incubation was practised at other shrines than those of Æsculapius, and especially in the temples of the Græco-Egyptian god Serapis. Before his altar at Babylon Alexander's generals slept for many nights during their master's last illness, vainly hoping that the god would reveal a remedy, for it was not always requisite that the patient himself should incubate, and at some places there were even professional dreamers who might be hired for the occasion. Other healing divinities were Apollo, Athene, and Hercules, to all of whom altars were erected in the great temple of Amphiaraus at Oropus. Here, where the prophet prince of Argos had been mysteriously swallowed by the earth, as he warred against Thebes, arose a sacred spring, and near it a shrine and oracle, where the patient might enjoy the advantage of a consultation of medical divinities. Having fasted three days from wine and one day from all food, he lay down to sleep on the fleece of a ram sacrificed to the deities. If the treatment indicated was successful, he gave thank-offerings and threw coins into the sacred well, and so famous for its cures did this temple at one time become that it is said to have ruined all the Asclepieia in Bœotia.

